# Evaluating HIV-1 Transmitted Drug Resistance and Clustering in Newly Diagnosed Patients in Romania (2019–2022)

**DOI:** 10.3390/v18010118

**Published:** 2026-01-15

**Authors:** Leontina Banica, Robert Hohan, Ionelia Nicolae, Raluca Patrascu, Corina Casangiu, Simona Paraschiv, Voichita Elena Lazureanu, Valerica Bica Profir, Dimitrios Paraskevis, Dan Otelea

**Affiliations:** 1 University of Medicine and Pharmacy “Carol Davila”, 020021 Bucharest, Romania; leontina.banica@umfcd.ro (L.B.); raluca.jipa@umfcd.ro (R.P.); 2 National Institute for Infectious Diseases “Prof. Dr. Matei Balș”, 021105 Bucharest, Romania; hohan.robert@gmail.com (R.H.); batan_ioana@yahoo.com (I.N.); corina.casangiu@gmail.com (C.C.); dotelea@mateibals.ro (D.O.); 3 The Research Institute of the University of Bucharest, 050663 Bucharest, Romania; 4 University of Medicine and Pharmacy “Dr. Victor Babeș”, 300041 Timișoara, Romania; lazureanu.voichita@umft.ro; 5 “Dr. Victor Babeș” Pneumophtysiology Infectious Diseases Hospital, 300310 Timișoara, Romania; valerica_bica@yahoo.com; 6 Department of Hygiene, Epidemiology and Medical Statistics, School of Medicine, National and Kapodistrian University of Athens, 11527 Athens, Greece; dparask@med.uoa.gr

**Keywords:** transmitted drug resistance, newly diagnosed, transmission networks, MSM, molecular clock

## Abstract

The HIV epidemic in Romania started in the late eighties with a large cohort of children nosocomially infected with subtype F1 strains, in parallel with sexual transmission. The purpose of the present study was to investigate the transmitted drug resistance (TDR), subtype distribution, and transmission clusters among persons diagnosed with HIV between 2019 and 2022 in Romania. The prototype of a person recently diagnosed with HIV in Romania is male, 20–50 years old, a late presenter, infected with F1, B, or A subtype. The rate of TDR varied over time, from 5% in 2019 to 15% in 2022. TDR affected mainly the first generation of NNRTIs and the PI class. The rate of late presentation was almost 60%, with 35% of persons qualifying as very late presenters. Subtype F1 is still preponderant in Romania, whereas other subtypes (B, A) and recombinants account for a quarter of HIV-1 new cases. Several transmission networks were identified in the study population, two of them associated with TDR in subtypes F1 and A1. The largest cluster consisted of 26 sequences, originating from Western Romania and introduced around 2007. Molecular clock analysis indicated different origin time points for different clusters, with the most recent in subtypes A1 and B, and the oldest in subtype F1. In conclusion, the HIV-1 epidemic in Romania is currently driven by sexual transmission, with MSM contribution continuously rising in recent years; there are also increases in TDR and the circulation of HIV-1 strains other than F1 (subtype B, A, recombinants).

## 1. Introduction

HIV-1 infections continue to be an important global health problem despite significant advances made in prevention and treatment. Combined antiretroviral therapy (cART) has lowered the mortality rates, increased life expectancy, and improved the quality of life. The current therapy is aiming to reduce the viral load below 200 copies/mL and, when possible, below the threshold of detection in order to achieve immune restoration and to prevent further sexual transmission [1]. Almost 40 million people were reported to live with HIV, and in 2023 77% were on cART, and 72% had suppressed viremia [2]. According to the Romanian HIV database, 18,768 people living with HIV (PLHIV) were registered at the end of 2024, of which 16,464 (87.7%) were under cART [3]. One epidemiological particularity of the Romanian HIV cohort is the large number of children nosocomially infected during early childhood (1987–1994). This cohort is quite unique, including a 1:1 male–female ratio of long-term survivors infected almost exclusively with subtype F1 [4]., This cohort currently accounts for more than 5000 patients that have been living for more than 35 years with the HIV-1 infection who have experienced multiple different therapies and accumulated over time several drug resistance mutations. Most of the patients are virologically suppressed, but there is still a risk of transmission of resistant strains when virological failure occurs. A decade ago, Romania faced an outbreak among intravenous drug users, the majority being HCV-coinfected and part of large HIV transmission networks [5].

The national surveillance of HIV cases is currently performed by the Department for Monitoring and Evaluation of HIV/AIDS infection in Romania [6]. Genotyping data of the circulating HIV-1 strains is meant to offer information about acquired and transmitted drug resistance (TDR), subtypes, and transmission networks identified among risk groups in newly diagnosed cases. Romania adheres to the European AIDS Clinical Society Guidelines [7] that recommend primary resistance testing before cART initiation. Due to financial restrictions, this could not be continuously and consistently implemented. Primary resistance is more often tested in seroconverters or presumably recently infected patients in whom resistance mutations may still be detected by Sanger sequencing. Besides its clinical significance for individual cases, monitoring transmitted drug resistance is also relevant for public health. Transmission of resistant viruses should be considered when designing and evaluating prevention strategies. TDR has an impact on both initial regimens and preexposure prophylaxis (PrEP). Currently, Romania is making its very first steps towards implementing a PrEP program [8]. The main risk population that would benefit from PrEP is gay, bisexual, and other men who have sex with men (MSM). A threefold increase in the number of HIV cases among this risk group was reported during the last ten years according to the national data [9]. The number could be even higher, as suggested by a recent publication that used phylogenetic analysis to estimate the route of transmission in Romanian HIV-1 subtype F1-infected patients [10].

Based on statistics from 2022, when diagnosing an HIV infection in the WHO European Region, there is a 50% probability that the patient will be a late presenter or have already experienced immune suppression (defined as a CD4 cell count of less than 350/µL). This can be partly explained by a slow recovery of public health services after the COVID-19 pandemic and an increase in regional population movement. Sequencing the viral genomes of newly diagnosed HIV infections can also be useful for detecting the emergence and spread of new subtypes and clades. This method can provide molecular epidemiology data to improve the understanding of geographical patterns of infection by investigating the characteristics of transmission networks. The sequences generated for testing drug resistance (*pol* gene segments encoding for the protease PR, reverse transcriptase RT, and integrase IN) can be used for those purposes. In this study, we have performed an analysis on TDR, subtype distribution, and transmission clusters identified in HIV-infected patients diagnosed between 2019 and 2022 in Romania. The results offer insights into the HIV-1 epidemic dynamics in Romania and may contribute to the design of targeted interventions in the near future.

## 2. Materials and Methods

### 2.1. Study Population

According to data from the Department for Monitoring and Evaluation of HIV/AIDS infection in Romania, between 2019 and 2022, a total of 2610 new HIV infections were reported in the country. Eligibility criteria in this study included confirmed HIV-1 seropositivity and absence of prior antiretroviral therapy. The study population included patients newly diagnosed with an HIV infection from 34 (out of a total of 41) counties across Romania. The patient samples were received and processed in the Molecular Diagnostics Laboratory of the National Institute for Infectious Diseases “Prof. Dr. Matei Bals”, which is the national reference center for HIV laboratory monitoring. Blood samples were collected from 424 newly diagnosed patients and tested for primary resistance. Clinical, laboratory testing, and epidemiological metadata were also collected and anonymized. The study was conducted according to the Declaration of Helsinki and approved by the Ethical Committee of the National Institute for Infectious Diseases “Prof. Dr. Matei Bals” (approval C09808/2024).

### 2.2. HIV-1 Genotyping

Sanger sequencing of the *pol* gene was undertaken by using the commercial ViroSeq HIV-1 Genotyping System (Celera Diagnostics, Alameda, CA, USA), which generated sequences that covered the complete PR gene and partial reverse transcriptase RT gene. For the integrase gene, DNA sequencing was performed by using the ViroSeq HIV-1 Integrase Genotyping Kit (Celera Diagnostics, Alameda, CA, USA). The instrument used for sequencing was the ABI 3500 Genetic Analyzer (Applied Biosystems, Foster City, CA, USA). The primary data were analyzed using the Sequencing Analysis Software Version 3.7 (Life Technologies), and the generated sequences were assembled with Seqscape Software v2.7 (Applied Biosystems, Foster City, CA, USA). After being manually checked in order to reach the complete or near-complete consensus sequences, 424 sequences corresponding to the PR and the RT region, and 249 integrase sequences were included in the analysis.

### 2.3. Surveillance Drug Resistance Mutations

All the sequences were checked for surveillance drug resistance mutations (SDRMs) in order to ascertain the proportion and rate of transmitted drug resistance (TDR) among new infections in Romania within the study period. This was performed by using the publicly available Calibrated Population Resistance (CPR) algorithm from the Stanford HIV-1 Resistance database, which checks its results against the list of Mutations for Surveillance of Transmitted HIV-1 Drug Resistance [11]. For the analysis, only the sequences covering both the reverse transcriptase and protease regions were included. Furthermore, based on the CPR quality analysis, the sequences that did not fulfill the algorithm’s quality criteria were removed. After the quality check, the remaining 408 (out of the initial 424) sequences were evaluated for reverse transcriptase and protease TDR, and 219 sequences for integrase TDR. All identified resistance mutations were annotated onto the phylogenetic trees to assess their distribution within transmission clusters. Moreover, all sequences were also screened for potential hypermutations using the Hypermut 3 algorithm [12].

### 2.4. HIV-1 Subtype Assignment

In order to determine the type of the infective HIV-1 strain, subtype, circulating recombinant form (CRF), or other variant, three online available tools were used (REGA HIV-1 Subtyping Tool—Version 3.46 [13], COMET HIV-1 2.4 [14], and the Geno2Pheno Virus Detection and Subtyping Tool [15]) to ensure consistent classification. The most prevalent subtypes, namely A, B, and F1, were used for further phylogenetic analysis.

### 2.5. Dataset Construction, Sequence Alignment, and Quality Control

Reference sequences were obtained from GenBank [16] and the Los Alamos National Laboratory (LANL) HIV Sequence Database [17]. Along with a complete dataset including all the study sequences, distinct datasets were constructed for each HIV-1 subtype identified among Romanian patients with recent HIV diagnosis (A, B, F1). The references were retrieved using NCBI BLAST in order to ensure the inclusion of the most similar sequences; duplicates were subsequently removed. To minimize phylogenetic bias, reference datasets were curated to ensure broad geographic representation and temporal diversity, incorporating the year and country of sampling as selection criteria.

All sequences were aligned using the AlignSeqs function in the DECIPHER package v3.4.0 within R v4.5.0 (RStudio 2024.12.1+563). Manual curation of the alignments was conducted using BioEdit v7.2.5 (Windows) and AliView v1.27 (macOS) to verify codon alignment and exclude problematic sequences.

### 2.6. Phylogenetic Inference and Molecular Clock Analysis

Both the maximum-likelihood (ML) and Bayesian approaches were used for performing phylogenetic analysis. ML trees were inferred using the FastTree software v2.1.11, whereas Bayesian analysis was performed using BEAST v2.7.7. For the latter, a strict molecular clock model was used, along with a Coalescent Exponential Growth tree prior and four gamma-distributed rate categories. Markov chain Monte Carlo (MCMC) chains were run for 11 million states with sampling every 1000 steps, yielding 10,000 trees after excluding an initial 10% burn-in. Log and trace files were examined in Tracer v1.7.2 to confirm convergence and effective sample sizes (ESS > 200). Because posterior and likelihood traces stabilized after approximately 6.5 million states, a conservative 70% burn-in was applied in TreeAnnotator v.2.7.7 to generate the final maximum clade credibility (MCC) tree. Phylogenetic trees were visualized using FigTree v1.4.4. Transmission clusters were defined as clades containing at least 3 study sequences with SH-like support higher than or equal to 0.9 and supported by a Bayesian posterior probability value of 1.0.

In addition to the primary tree that includes all the pure sequences, subtype-specific ML and Bayesian trees were constructed using the same pipeline. In order to provide adequate phylogenetic context within each clade, the resulting phylogenetic trees were rooted using different subtype sequence references: for the subtype A tree, we used subtype G references; for the subtype B tree, we used subtype D references; and for the subtype F1 tree, we used references of the F2 subtype. All the reference sequences used in this analysis were provided as Supplementary File S1.

### 2.7. Statistical Analysis

Both chi-square tests and Fisher’s exact test were employed to evaluate potential associations between demographic, clinical, and epidemiological characteristics and the prevalence of surveillance drug resistance mutations (SDRMs) among newly diagnosed HIV patients. In order to quantify the level of agreement between the three HIV-1 subtyping algorithms, both Cohen’s Kappa and Fleiss’ Kappa statistics were calculated using R version 4.5.0.

## 3. Results

### 3.1. Characteristics of the Study Population

The number of newly diagnosed patients recruited during the study period varied widely, mainly due to the COVID pandemic and the consequent fluctuations in addressability and access to HIV testing. The number of patients included in this analysis decreased from 198 patients in 2019 to 51 in 2020. The number of newly diagnosed patients included in 2021 and 2022 slowly increased but did not reach the pre-pandemic level (Table 1).

The majority of the individuals newly diagnosed with HIV-1 during 2019–2022 included in this study were male (80.7%) between 20 and 50 years old (363/424, 85.6%), most of them being diagnosed as late presenters (CD4 < 350 cell/µL) (59.6%) or even very late presenters (35.1%) (CD4 < 200 cell/µL) (Table 1). The age distribution follows the Gaussian curve with the peak in the group of 31–40 years old patients (34.9%), followed by the group of 21–30 years old patients (27.6%) and 41–50 years old patients (23.1%).

Most of these patients reported having acquired HIV infection by the heterosexual route, followed by MSM and people who inject drugs (PWID). Three cases of mother-to-child transmission were reported in 2021. The demographics of the population, as well as the routes of transmission, are similar to overall national data [3]. Supplementary Figure S1 presents the comparison between the proportion of self-reported transmission routes among HIV new cases in our study versus national reports during the period 2019–2022.

### 3.2. Subtyping Analysis

The analysis performed with the three distinct tools showed several discrepancies between the outputs: out of 424 sequences evaluated, 301 had a fully concordant subtype assignment with all methods; in 417 cases, a consensus between at least two subtyping tools was observed, whereas for 7 sequences, the subtype assignment results were different across all three algorithms used. The subtype assessment based on a consensus approach (minimum two concordant results generated with the used subtyping tools) is presented in Table 1.

Pairwise Cohen’s Kappa analysis showed substantial agreement between REGA and geno2pheno (κ = 0.743) and between geno2pheno and COMET (κ = 0.625), but only moderate agreement between COMET and REGA (κ = 0.517). Despite this, Fleiss’ Kappa revealed low overall agreement across all three tools (κ = 0.060), likely due to subtype imbalance and inconsistent calls for rare subtypes. Standardized residuals from a chi-square test identified specific subtype-algorithm biases, notably COMET overcalling CRF (uncertain), REGA favoring A1, and geno2pheno aligning most closely with the majority of F1 subtype sequences.

Using Fleiss’ Kappa statistical test, we found a significant association between subtype F1 and self-reported heterosexual route of transmission, while subtypes B and A1 were linked to self-reported MSM transmission (Supplementary Figure S2). In addition to these preliminary results, the phylogenetic analysis contributed important insights into the HIV-1 subtypes distribution and transmission clusters, as described in the next Section 3.4.

### 3.3. Transmitted Drug Resistance

**Figure 1:**
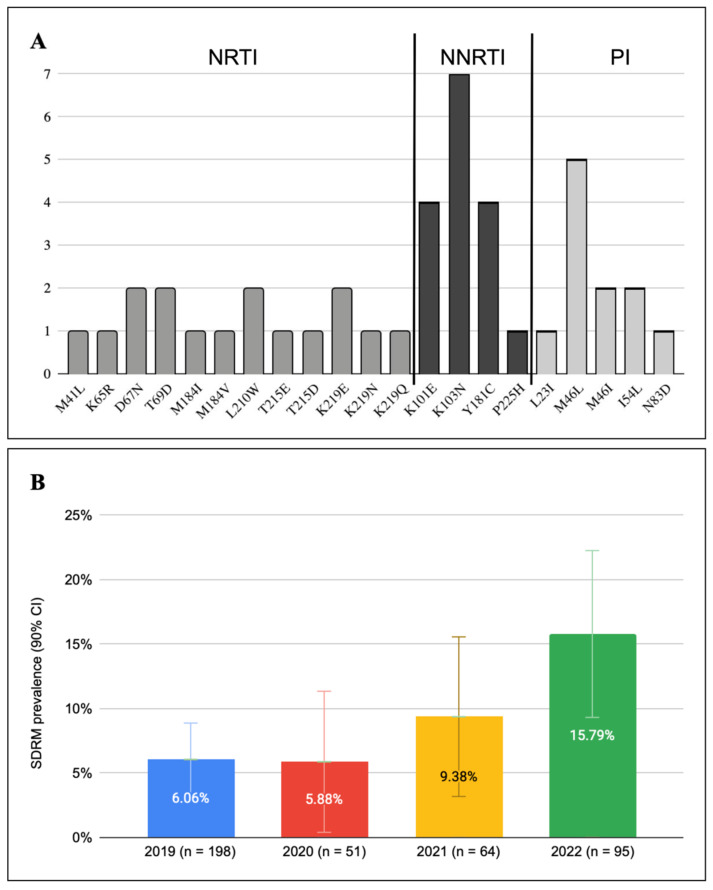
(**A**). Summary of mutations identified, ordered by their AA position in the *pol* gene. (**B**). TDR distribution by year of HIV-1 diagnosis. Bars indicate the 90% confidence intervals for annual TDR estimates.

By evaluating PR and RT sequences from Romanian patients diagnosed with HIV during 2019–2022, a total of 36 sequences (8.82%) were found to harbor SDRMs. Of those identified with RT and PR mutations, 30 sequences (7.69%) had one mutation, while viral genomes with 2 and 3 SDRMs were found in the other six. A detailed description of identified SDRMs is provided in Figure 1, while Table 2 presents the characteristics of the viral sequences with multiple SDRMs.

The most frequent SDRM was K103N (7 sequences) that strongly impacts the first generation of non-nucleoside reverse transcriptase inhibitors (NNRTIs), followed by the M46L protease mutation (5 sequences), a mutation associated with reduced susceptibility to atazanavir (ATV) and lopinavir (LPV) protease inhibitors (PIs) (Figure 1).

Thymidine analog mutations (TAMs), involved in resistance to nucleoside reverse transcriptase inhibitors (NRTIs), specifically stavudine (d4T) and zidovudine (AZT), were identified among the analyzed sequences. Both TAM pathways were present: TAM-1 (M41L + T215D) and TAM-2 (D67N + K219N/Q/E).

Other PI SDRMs were identified, such as L23I, I54L, and the N83D mutation that reduces the susceptibility to LPV and DRV.

In terms of transmitted drug resistance by classes, similar prevalence was observed: 3.2% (n = 13) for NRTIs, 3.7% (n = 15) for NNRTIs, and 2.9% (n = 12) for PIs.

For the IN region, of 219 sequences available, only one sequence presented an SDRM, namely the E138K mutation, corresponding to a 0.46% rate of TDR to the integrase inhibitors (INSTI) class. The sequence had no mutations in the RT/PR regions and belonged to a patient diagnosed in 2021 with a good immunologic status (CD4 = 727 cells/µL) and without co-infections. The patient was infected with subtype F1 and reported acquiring HIV by MSM sexual contact. E138K is a common accessory resistance mutation and was reported in patients receiving RAL, EVG, CAB, and DTG. This mutation does not reduce susceptibility to INSTI itself. No major mutations were detected in the analyzed integrase sequences.

In this study, 32 (7.8%) sequences had SDRMs for only one class of antiretroviral drugs, while TDR for two different classes was registered in four sequences (0.98%). In fact, four out of the six HIV-1 sequences with multiple SRDMs had TDR to multiple classes, suggesting more recent transmission events from individuals with multiple drug resistance patterns.

The rate of TDR varied slightly between subtypes, from 5.35% (3/56) in subtype B, 9.5% (28/296) in subtype F1, to 23.5% (4/17) in subtype A1/A6 sequences. However, given the rather small proportion of the non-F1 proportion of subtypes, this observation has limited relevance. Overall, a significant increase in SDRM’s prevalence over the four-year timeline was observed, starting from 6.06% in 2019 to more than 15% in 2022 (χ^2^ = 8.18, *p* = 0.042; Figure 1B).

### 3.4. Transmission Networks Identified Among Newly Diagnosed HIV Patients

**Figure 2:**
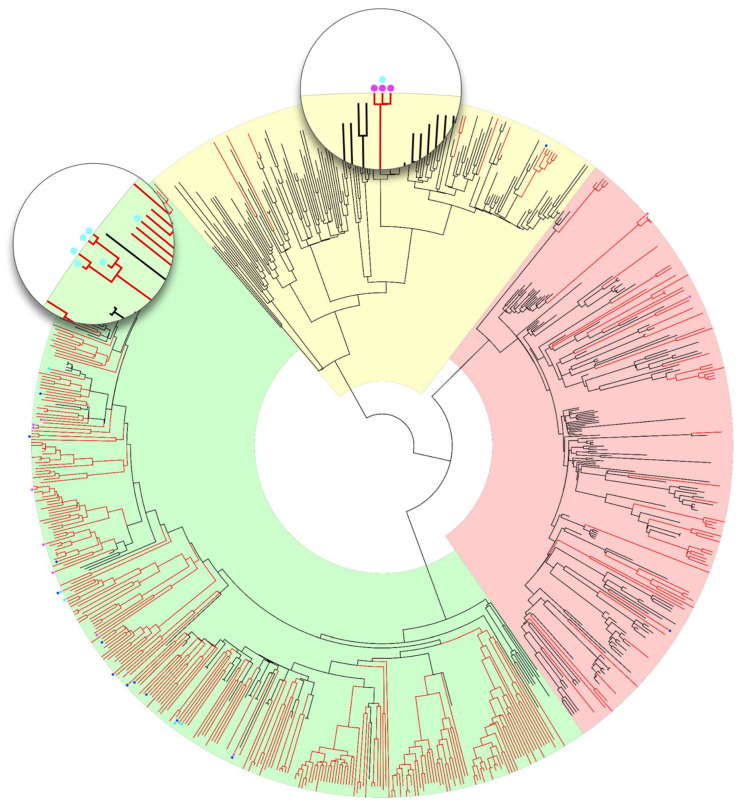
Bayesian phylogenetic tree. Branch colors: black (references from GenBank) and red (Romanian newly diagnosed cases during 2019–2022). Subtypes are highlighted as yellow (subtype A), red (subtype B), and green (subtype F1). TDR mutations (dots): blue (NRTIs), magenta (NNRTIs), and cyan (PIs). Zoomed-in windows show two clusters of sequences with a high degree of TDR.

In order to assess more precisely the relatedness between viral strains from newly diagnosed patients (especially those with SDRMs) and other similar HIV-1 sequences, a phylogenetic analysis was performed using Bayesian analysis. The resulting phylogenetic tree consisted of 784 sequences (424 Romanian HIV sequences analyzed in this study and 360 references of the most prevalent subtypes in Romania, namely F1, B, and A1, A6) and is represented in Figure 2.

Molecular clock analysis was consequently performed on separate subtype datasets in order to determine more accurately the time to the most recent common ancestors (tMRCA) for the identified clusters.

Transmission cluster analysis performed on subtype-specific phylogenetic trees indicated the following results: two well-supported subtype A (A1 and A6) clusters, 8 clusters of subtype B, and 23 clusters of subtype F1 sequences were identified among the study population. We also identified 14 dyads (pairs of sequences found to have a close phylogenetic relationship), broken down by subtype as follows: subtype A (1), subtype B (3), and subtype F1 (10).

**Figure 3:**
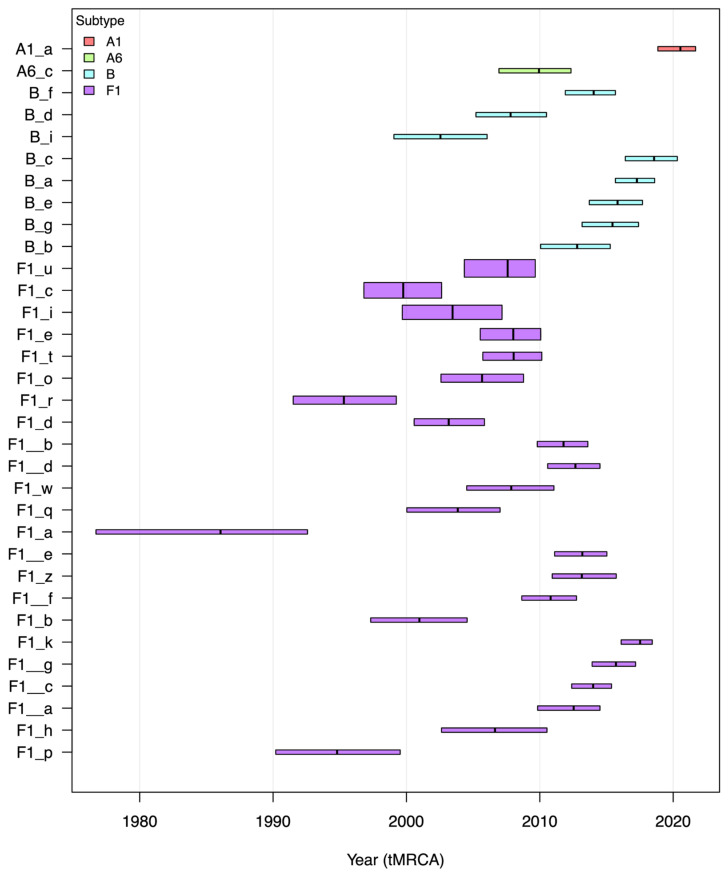
Temporal depth and transmission timing across subtypes A, B, and F1 in Romania. Each horizontal bar represents an individual cluster, plotted by its mean time to the most recent common ancestor (tMRCA) and corresponding 95% highest posterior density (HPD) interval. Bar heights are proportional to the cluster size. Clusters are arranged along the y-axis by subtype and cluster size, with the x-axis indicating calendar year.

Figure 3 illustrates the subtype-specific transmission clusters, the mean tMRCA, and the corresponding 95% highest posterior density (HPD) interval.

Phylogenetic and phylodynamic analyses results showed that the majority of HIV sequences from newly diagnosed persons in Romania were part of well-supported phylogenetic clusters (57%, 242 out of 424). There were 23 transmission clusters identified within the F1 subtype sequences and 10 others with non-F1 subtype (8 with subtype B, one with A1, and each with A6). The cluster with the more recent tMRCA belongs to subtype A1, estimated to around 2020 (cluster A1_a in Figure 3), whereas the one with the oldest tMRCA belongs to subtype F1 and was dated back around 1986 (cluster F1_a in Figure 3). As expected, the highest number of clusters was identified within subtype F1; the most recent cluster was estimated around 2017 (cluster F1_k). The mean tMRCA for subtype B transmission clusters ranged between 2003 and 2019. The largest transmission cluster consists of 26 subtype F1 sequences (cluster F1_u), corresponding mainly to young male patients who share a geographical region in the west of Romania. Cluster age uncertainty, as reflected by the 95% highest posterior density (HPD) intervals, varied substantially across transmission clusters, ranging from 2.6 to 11.3 years. The widest temporal uncertainty was observed in older clusters, such as F1_l (11.3 years) and F1_f (7.7 years), consistent with their deeper genetic divergence and potentially incomplete sampling of intermediate transmission links. In contrast, more recent clusters, including F1__c (2.6 years) and A_a (2.7 years), exhibited narrower HPD intervals, indicating stronger temporal resolution and more recent common ancestry among sampled individuals. These tighter intervals may reflect more contemporaneous transmission events and higher sampling density during the recent epidemic phase.

### 3.5. Subtype A1 Cluster of MSM-Associated Sequences with Shared K103N NNRTI Resistance

Two clusters showed notable patterns of transmitted drug resistance. The first cluster, distinctly marked in the tree with magenta dots (Figure 2), comprised three partial genomes of subtype A1, all of them presenting the K103N mutation that affects the first generation of NNRTIs. One of these sequences additionally possessed the I54L protease mutation (marked with both cyan and magenta dots). The most recent common ancestor for this cluster was estimated in 2020.54 (2018.85–2021.68), suggesting very recent transmission. The corresponding patients were male, aged 22, 25, and 28 years, and reported sex with men as the probable way of transmission. All three were diagnosed in 2022. The uniform diagnosis year, narrow age range, consistent transmission route, and shared resistance profile suggest this is a recent transmission network among MSM.

### 3.6. Subtype F1 Transmission Cluster with PI-Associated TDRMs

The second cluster with TDR included five F1 sequences with PI-associated TDRMs, namely the M46L mutation. No major resistance mutations were detected for reverse transcriptase or integrase inhibitors. The cluster included four males and one female diagnosed between 2019 and 2022, aged 20 to 56 years. Three males (ages 20, 33, and 56) self-identified as men who have sex with men (MSM), while one male (age 52) and the female (age 20) reported heterosexual transmission. The most common recent ancestor was dated back around 2018, consistent with a longer circulation of this resistant variant.

## 4. Discussion

At the end of 2024, Romania reported 18,768 PLHIV, mainly concentrated in the Bucharest metropolitan area (more than 10,000). Currently, the epidemic is driven by sexual transmission, mostly in the young and middle-aged male population (between 20 and 50 years old), often diagnosed as late presenters. By analyzing samples collected during 2019–2022 from newly HIV diagnosed persons, this study showed that MSM transmission was acknowledged in one-third of the new HIV cases. An increase in the HIV cases among MSM was reported in recent years, from about 20% of newly diagnosed cases in 2017 to over 30% in 2024 [9] (Supplementary Figure S1). The geographical origin of the sequenced strains was in 34 out of the 41 counties in Romania, but predominantly from the Bucharest metropolitan area and Timis Regional Center, the two hotspots for HIV-1 cases, with more than half of PLHIV located here [9]. The increase in HIV and other sexually transmitted infections (STIs) in MSM was more recently linked to chemsex [18]. In this study, 35.1% of the patients diagnosed between 2019 and 2022 declared themselves as part of the MSM/Bisexual population. Previous analyses performed in Romania on newly diagnosed patients infected with the F1 subtype suggested that MSM transmission might be higher (double) than the self-reported one [10]: around 70% of the sequences from newly diagnosed persons were part of a MSM transmission chain.

When compared to Western European countries, where PrEP is available for populations at high risk of being infected with HIV and has led to a reduction in HIV transmission [19], Romania is just about to start a PrEP program. However, some of the individuals at risk (e.g., MSM) are already purchasing it from online markets. The advantage of PrEP usage in reducing transmission seems to outweigh the risk of selecting drug-resistant mutations [20]. Several studies suggested that DR reported in persons under PrEP was not a matter of transmission, but of selection under suboptimal drug levels [21]. In our study, K65R and M184V/I, mutations that were reported in persons infected while under PrEP, were found in a limited number of sequences. The most frequent SDRM was K103N, followed by M46L/I protease mutation and different TAMs, mutations that are selected exclusively by antiretroviral therapy. These mutations are induced mainly by older generations of antiretroviral drugs (which are in limited or no use at this moment), but they can persist due to their low impact on viral fitness.

The rate of TDR showed a significantly increasing trend during the study period, rising from 6% in 2019 to 15% in 2022. Although further data are needed to confirm this trend, it should be noted that similar TDR rates were reported by other Central European countries, suggesting a possible rising concern for this region [22]. The INSTI class was the least affected by TDR, with no impact on the current first-line regimens based on the second generation of INSTI. However, a future increase in the TDR rate to this ARV class cannot be excluded due to its extended use [23]. The identified SDRMs affected the first generation of NNRTI drugs, leaving for the moment open the use of the second generation.

In accordance with previous studies that indicated higher TDR rates in HIV subtype A-infected persons [24,25], the highest TDR (23.5%) was observed in subtype A1/A6-infected patients. However, due to the limited number of subtypes A1 and A6 identified in this study, the significance of our observations is unclear.

Another important factor that might impact HIV transmission is late diagnosis: 59.6% of the subjects in this study were diagnosed as late presenters (CD4 < 350 cells/µL), and more than a third had a more advanced disease (CD4 < 200 cells/µL or AIDS defining conditions). Due to the fitness impact of some TDMs, the virus tends to return to the wild type.

A hallmark of Romanian HIV-1 epidemics was the high prevalence of subtype F1: 93% of PLHIV in the 1990s were infected with this viral subtype [26]. In accordance with the founder effect hypothesis, the present study showed that the F1 subtype continues to be the most prevalent, accounting for more than 70% of new cases. It should be noted that the incidence of subtype F1 viruses observed in this study is lower than in previous reports [27]. Other subtypes were identified among newly diagnosed persons, namely subtype B (15%), subtype A1 and A6 (4%), as well as recombinant forms (more than 5%). An increase in the worldwide proportion of subtype A and C infections was reported in recent years, while the recombinant forms decreased [28]. The data presented here are a suggestive representation of local HIV dispersal, mainly influenced by increasing population mobility; the contribution of the war in Ukraine to the spread of subtype A6 viruses was also acknowledged in other countries [29].

Phylogenetic analysis findings underscore the dominant and persistent role of subtype F1 in the Romanian HIV epidemic. Subtype F1 not only accounted for the majority of identified clusters but also showed evidence of both long-standing and ongoing transmission, spanning nearly three decades of inferred evolutionary history. This pattern highlights its deep entrenchment within the population and continued circulation through diverse transmission networks. In contrast, subtypes B and A were represented by fewer clusters of generally smaller size, with tMRCAs to be dated later in time, suggesting more recent introductions or localized outbreaks. Although transmission within these subtypes remains active, particularly among key populations, their overall clustering pattern suggests more episodic and geographically or behaviorally bounded spread relative to subtype F1.

An important issue raised by this study is the HIV-1 subtype assessment comparison between several available subtyping tools. When compared with the phylogenetic analysis, the COMET software seems to underestimate the F1 subtype, overcalling CRFs (uncertain), while giving good results in differentiating between subtype A1 and A6. The REGA tool was unable to differentiate between subtype A1 and A6, as previously reported [30]. The geno2pheno new subtyping algorithm [15] aligned most closely with the majority of subtype F1 and was able to identify subtype A1/A6 or recombinant forms and thus, in this case, returned the closest results. Supplementary Table S1 presents the strengths and limitations of each subtyping tool used in this study. These findings underscore both the strengths and limitations of multi-tool subtyping approaches in the genetically diverse HIV-1 epidemic and underline the importance of using phylogenetic approaches to improve the characterization of circulating HIV strains. Previous studies from Romania associated subtype B mainly with MSM transmission, while subtype F1 was transmitted mainly through heterosexual contact and parenterally (nosocomial or injected drug use) [27,31,32]. In this study, which was performed on more recent HIV cases, MSM transmission networks were also identified among persons infected with subtype F1. The largest transmission cluster was identified among F1-infected patients, consisting of 26 sequences from 25 males originating from the west of Romania. In line with previous findings that suggested underreporting of MSM behavior in Romania [10], we can assume this was an MSM-associated transmission network. Similarly, the identified TDR-associated cluster formed by five sequences (three MSM) is characterized by a shared PIs resistance profile (M46L). The presence of a heterosexual female within this cluster suggests a transmission chain between MSM and heterosexuals.

We acknowledge several limitations of this study. The sampling, while matching the demographics of the persons newly diagnosed with HIV-1 in Romania, was limited in number and mainly focused on two main geographical regions (metropolitan Bucharest area and Timis County). It is worth mentioning that during the study period, more than one-third of new HIV-1 cases were reported in these areas [6]. The sampling varied widely during the study period, mainly due to HIV underdiagnoses associated with the COVID pandemic. The self-reporting transmission data might have been influenced by stigma, especially in the Gay, Bisexual, and Other Men who have Sex with Men (GBMSM) population. The genomic region used in this analysis (*pol* gene) did not allow the full characterization of recombinant forms and might have underestimated the number of URFs.

## 5. Conclusions

Transmitted drug resistance is seemingly increasing and thus continues to be a matter of concern in Romania. The HIV-1 epidemic is currently driven by sexual transmission, with MSM contribution continuously rising in recent years, as well as the rate of TDR and the circulation of other HIV-1 strains than F1 (subtype B, A6, recombinants). Although the current TDR has a limited impact on the first-line therapies, the increasing trend needs continuous monitoring. Phylogenetic analysis proved to be useful when subtyping with the different available tools returned discordant results, and in characterizing the viral spread in this population.

## Figures and Tables

**Table 1:** Study population characteristics.

		TDR [Drug Class]			Any
	No of Patients	*n* [NNRTIs]	*n* [NRTIs]	*n* [PIs]	*n* (%)
Year					
2019	198	5	6	3	12 (6.06%)
2020	51	0	2	1	3 (5.88%)
2021	64	3	1	2	6 (9.37%)
2022	95	7	4	6	15 (15.79%)
					
Gender					
Male	342	12	10	9	28 (8.18%)
Female	82	3	3	3	8 (9.75%)
					
Age (years)					
≤10	3	0	0	0	0
11–20	17	1	0	2	3 (17.6%)
21–30	117	11	8	2	18 (15.3%)
31–40	148	1	5	7	12 (8.1%)
41–50	98	2	0	0	2 (2%)
51–60	25	0	0	1	1 (4%)
>61	16	0	0	0	0
					
Transmission route					
HET	231	9	6	6	19 (8.22%)
MSM	149	6	7	6	17 (11.4%)
PWID	31	0	0	0	0
MTCT	3	0	0	0	0
N/A	10	0	0	0	0
					
CD4 counts (cells/µL)					
<200	149	4	8	4	14 (9.39%)
200–350	104	3	2	2	7 (6.73%)
351–500	73	4	0	2	5 (6.8%)
>500	82	4	2	3	9 (10.9%)
N/A	16	0	1	1	1 (6.2%)
					
Subtype					
A1	6	3	0	1	3 (50%)
A6	11	0	1	0	1 (9%)
B	64	2	1	0	3 (4.6%)
C	9	1	0	0	1 (11%)
F1	310	9	11	11	28 (9.03%)
G	1	0	0	0	0
CRFs	12	0	0	0	0
CRFs (uncertain)	3	0	0	0	0
URFs	8	0	0	0	0

N/A—not available.

**Table 2:** Characteristics of HIV-1 sequences presenting multiple SDRMs.

Viral Subtype	seq_ID	Sampling Year	Patient Gender	Drug Class											Total Mutations
				NRTI				NNRTI			PI				
A	2684	2022	M					K103N					I54L		2
F1	2418	2019	F								L23I	M46I			2
F1	4431	2019	F	M41L		T215D									2
F1	1465	2022	M		D67N		K219N							V82F	3
F1	4592	2019	F		D67N		K219Q							V82A	3
F1	5068	2019	M				K219E		Y181C	P225H					3

## Data Availability

The sequences analyzed in this study have the following accession numbers in GenBank: subtype F1 (PQ512891-PQ513202), subtype A (PX443660-PX443676), subtype B (PX443677-PX443740), and others (PX443677-PX443740).

## Supplementary Materials

The following supporting information can be downloaded at: https://www.mdpi.com/article/10.3390/v18010118/s1. Figure S1: Timeline overview for the studied period (2019–2022) regarding the self-reported route of transmission for new HIV diagnoses. Shown are the percentage proportions for both the national (Romania) data and the patients seen at the National Institute for Infectious Diseases. Only the top 3 pathways are shown, as they cover >95% of cases: heterosexual (HET), men who have sex with men (MSM), people who inject drugs (PWID); Figure S2: Summary of the statistical results of Fleiss’ Kappa test regarding the correlation between the self-reported route of transmission and the results of HIV sequence subtyping (as determined by the consensus of the 3 algorithms used in this study: geno2pheno, COMET and REGA). Illustrated are the standardized residuals with the *p*-values in parentheses for all possible combinations tested, with over-represented situations colored in red and under-represented ones in blue; Table S1: Comparison between HIV-1 subtyping tools. Across 424 sequences, 301 were fully concordant across COMET/REGA/geno2pheno and 417 had ≥2-tool consensus; discordance was concentrated in rarer categories under a strongly F1-skewed subtype distribution. COMET is highly scalable and conservative but more frequently returns “uncertain/CRF (uncertain)” calls, reducing direct comparability. REGA provides phylogeny/recombination-aware subtyping but can differ in how A-lineages and recombinants are labelled. geno2pheno showed the strongest overall alignment with the other two tools (highest pairwise κ), making it useful as an adjudicator in a 2-of-3 consensus framework, but category definitions/reporting may not map perfectly across tools; File S1: The alignment of reference sequences used in this study (fasta file format).

## Abbreviations

The following abbreviations are used in this manuscript:

MSM	Men who have sex with men
TDR	Transmitted drug resistance
SDRM	Surveillance of drug resistance mutations
BEAST	Bayesian evolutionary analysis sampling trees
tMRCA	Time to the most recent common ancestor
CRF	Circulating recombinant form
URF	Unique recombinant form
PrEP	Pre-exposure prophylaxis
NRTI	Nucleosidic reverse transcriptase inhibitor
NNRTI	Non-nucleosidic reverse transcriptase inhibitor
PI	Protease inhibitor
