# Supplementary material for: Evaluating HIV-1 Transmitted Drug Resistance and Clustering in Newly Diagnosed Patients in Romania (2019–2022)
Source: Viruses. 2026 Jan 15;18(1):118. doi: 10.3390/v18010118 (PMC12846587; doi:10.3390/v18010118)
Supplement: Supplementary file 1 [file viruses-18-00118-s001.zip › viruses-4073815-supplementary/Supplementary Figure S1.pdf]

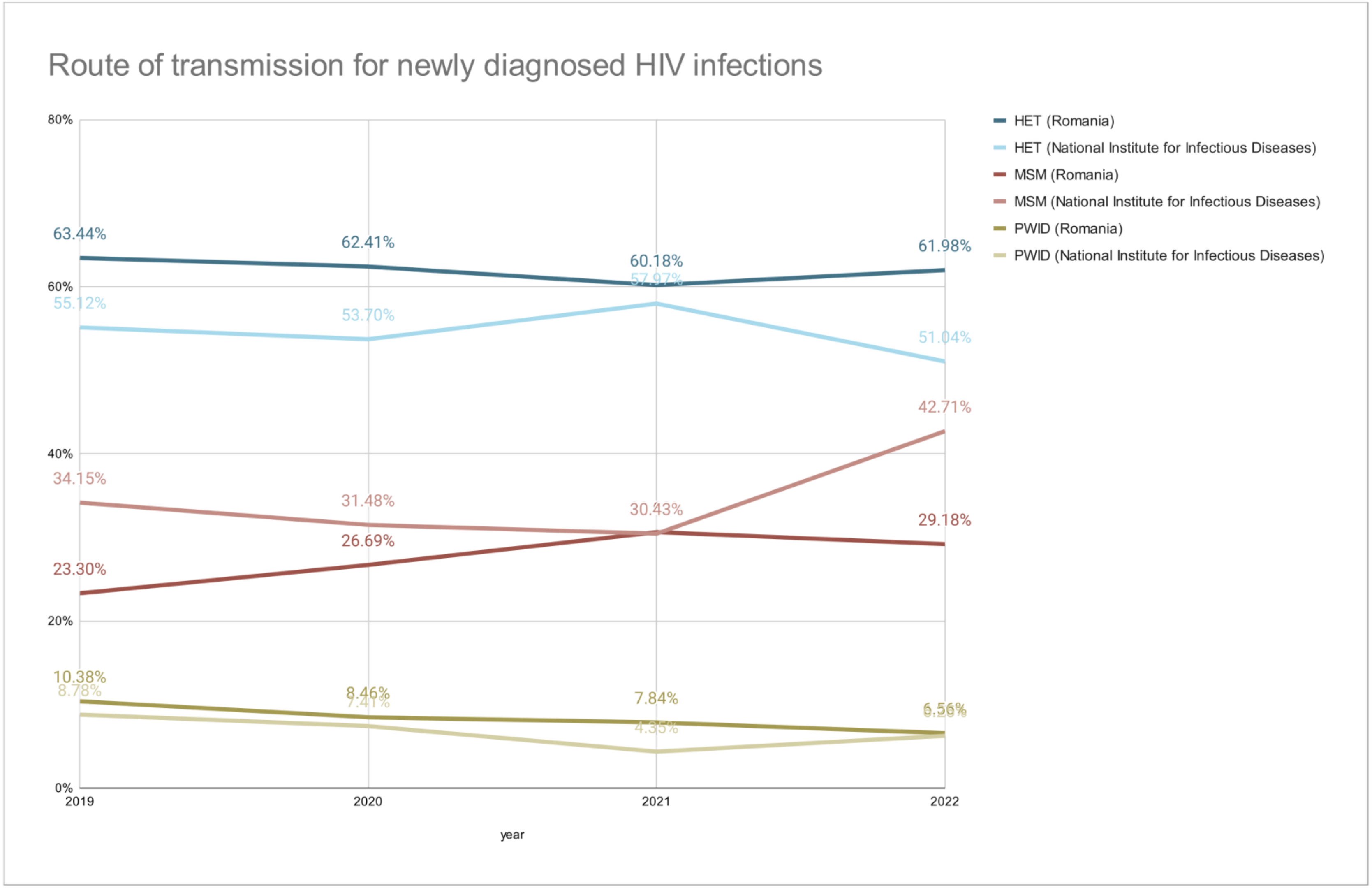

Figure S1. Timeline overview for the studied period (2019-2022) regarding the self-reported route of transmission for new HIV diagnoses. Shown are the percentage proportions for both the national (Romania) data and the patients seen at the National Institute for Infectious Diseases. Only the top 3 pathways are shown, as they cover >95% of cases: heterosexual (HET), men who have sex with men (MSM), people who inject drugs (PWID).
