# Supplementary material for: Evaluating HIV-1 Transmitted Drug Resistance and Clustering in Newly Diagnosed Patients in Romania (2019–2022)
Source: Viruses. 2026 Jan 15;18(1):118. doi: 10.3390/v18010118 (PMC12846587; doi:10.3390/v18010118)
Supplement: Supplementary file 1 [file viruses-18-00118-s001.zip › viruses-4073815-supplementary/Supplementary Figure S2.pdf]

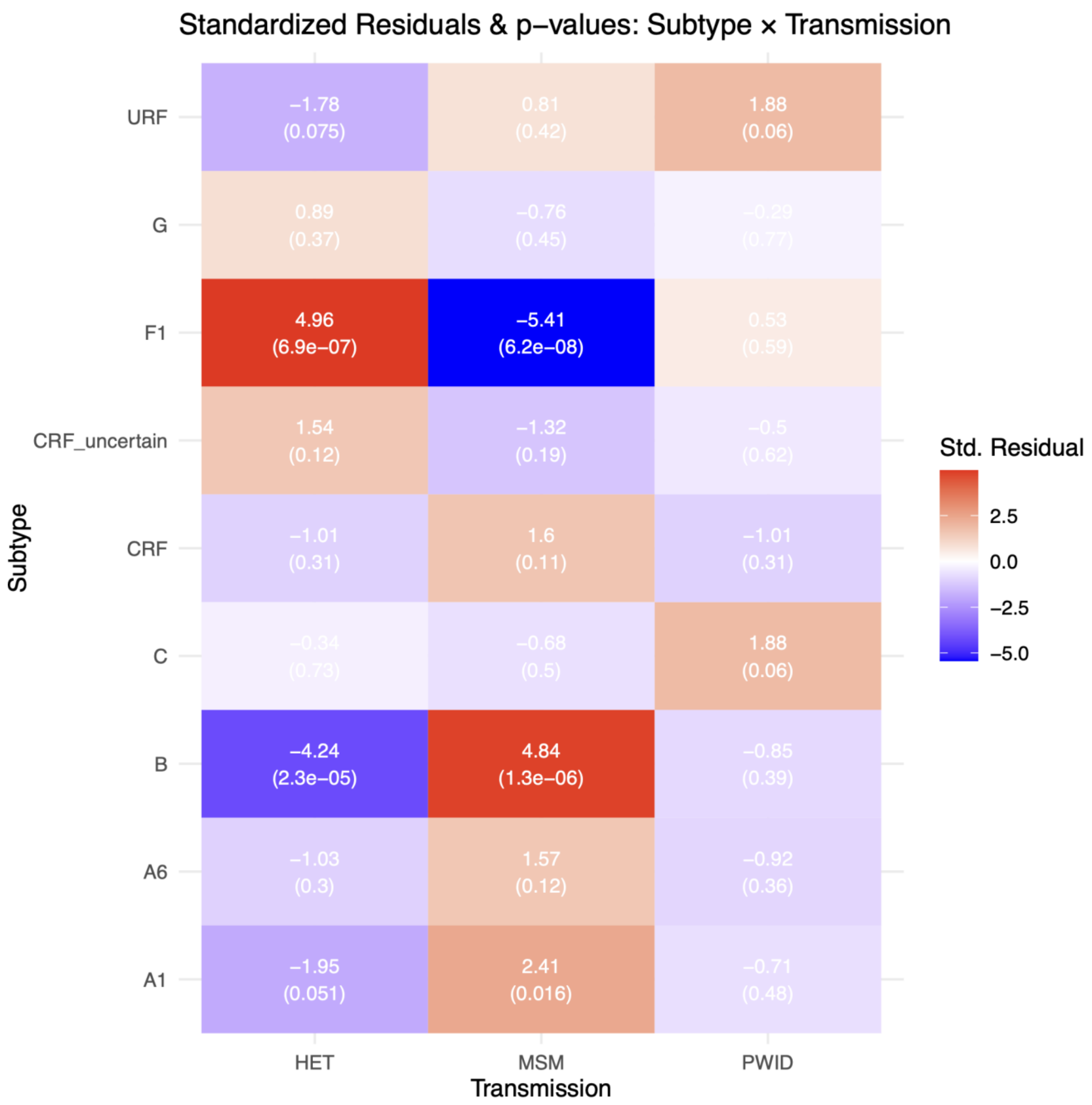

Figure S2. Summary of the statistical results of Fleiss' Kappa test regarding the correlation between the self-reported route of transmission and the results of HIV sequence subtyping (as determined by the consensus of the 3 algorithms used in this study: geno2pheno, COMET and REGA). Illustrated are the standardized residuals with the p-values in parentheses for all possible combinations tested, with over-represented situations colored in red and under-represented ones in blue.
