# Supplementary material for: Evaluating HIV-1 Transmitted Drug Resistance and Clustering in Newly Diagnosed Patients in Romania (2019–2022)
Source: Viruses. 2026 Jan 15;18(1):118. doi: 10.3390/v18010118 (PMC12846587; doi:10.3390/v18010118)
Supplement: Supplementary file 1 [file viruses-18-00118-s001.zip › viruses-4073815-supplementary/Supplementary Table S1.pdf]

Supplementary Table S1. Comparison between HIV-1 subtyping tools. Across 424 sequences, 301 were fully concordant across COMET/REGA/geno2pheno and 417 had ≥2-tool consensus; discordance was concentrated in rarer categories under a strongly F1-skewed subtype distribution. COMET is highly scalable and conservative but more frequently returns “uncertain/CRF (uncertain)” calls, reducing direct comparability. REGA provides phylogeny/recombination-aware subtyping but can differ in how A-lineages and recombinants are labelled. geno2pheno showed the strongest overall alignment with the other two tools (highest pairwise κ), making it useful as an adjudicator in a 2-of-3 consensus framework, but category definitions/reporting may not map perfectly across tools.

Tool	Strengths	Limitations	Pattern seen in our 424 sequences
**COMET**	- very fast and scalable<br>- consistent automated subtype calls<br>- readily flags ambiguous/recombinant-like sequences	- tends to be conservative and may over-label ambiguity<br>- “uncertain/CRF (uncertain)” categories can reduce comparability with other tools	- more **CRF (uncertain)** and fewer **F1** assignments than the others<br>- lowest agreement with REGA (κ = 0.517)
**REGA**	- phylogeny- and recombination-aware (bootscan/decision rules)<br>- widely used in surveillance workflows<br>- informative outputs beyond a single label	- subtype designation can differ for A-lineages and rarer categories depending on reference set/rules<br>- recombinant labeling not always harmonized across tools	- strong agreement with geno2pheno (κ = 0.743)<br>- relatively more **A1/URF** and fewer **A6** calls
**geno2pheno**	- good adjudicator in consensus pipelines<br>- generally aligns with both other tools<br>- useful for cross-checking borderline calls	- decision logic less transparent than REGA<br>- fewer explicit “uncertain” labels, which can shift counts across categories	- most aligned overall (κ = 0.743 with REGA; κ = 0.625 with COMET)<br>- relatively more **F1/A6**, rarely “CRF (uncertain)”
